# Combining fish and benthic communities into multiple regimes reveals complex reef dynamics

**DOI:** 10.1038/s41598-018-35057-4

**Published:** 2018-11-16

**Authors:** Mary K. Donovan, Alan M. Friedlander, Joey Lecky, Jean-Baptiste Jouffray, Gareth J. Williams, Lisa M. Wedding, Larry B. Crowder, Ashley L. Erickson, Nick A. J. Graham, Jamison M. Gove, Carrie V. Kappel, Kendra Karr, John N. Kittinger, Albert V. Norström, Magnus Nyström, Kirsten L. L. Oleson, Kostantinos A. Stamoulis, Crow White, Ivor D. Williams, Kimberly A. Selkoe

**Affiliations:** 1 Department of Biology, University of Hawai’i at Mānoa, Honolulu, HI 96822 USA; 2 Hawai‘i Institute of Marine Biology, University of Hawai‘i at Mānoa, Kaneohe, HI 96744 USA; 3 Pristine Seas, National Geographic Society, Washington D.C, 20036 USA; 4 Department of Natural Resources and Environmental Management, University of Hawai‘i at Mānoa, Honolulu, HI 96822 USA; 5 Office of National Marine Sanctuaries, National Oceanic Atmospheric Administration, Honolulu, HI 96818 USA; 6 Stockholm Resilience Centre, Stockholm University, Stockholm, Sweden; 7 Global Economic Dynamics and the Biosphere Academy Programme, Royal Swedish Academy of Sciences, Stockholm, Sweden; 8 School of Ocean Sciences, Bangor University, Anglesey, LL59 5AB UK; 9 Center for Ocean Solutions, Stanford University, Stanford, CA 94305 USA; 10 Lancaster Environment Centre, Lancaster University, Lancaster, LA1 4YQ UK; 11 Ecosystem Sciences Division, Pacific Islands Fisheries Science Center, National Oceanic Atmospheric Administration, Honolulu, HI 96818 USA; 12 National Center for Ecological Analysis and Synthesis, University of California Santa Barbara, Santa Barbara, CA 93101 USA; 13 Oceans Program, Environmental Defense Fund, San Francisco, CA 94105 USA; 14 Center for Oceans, Conservation International, Honolulu, HI 96825 USA; 15 Julie Ann Wrigley Global Institute of Sustainability, Arizona State University, Tempe, AZ 85281 USA; 16 Curtin University, Bentley, WA 6102 Australia; 17 Department of Biological Sciences, California Polytechnic State University, San Luis Obispo, CA 93407 USA; 18 Hopkins Marine Station, Stanford University, Monterey, CA 93950 USA

## Abstract

Coral reefs worldwide face an uncertain future with many reefs reported to transition from being dominated by corals to macroalgae. However, given the complexity and diversity of the ecosystem, research on how regimes vary spatially and temporally is needed. Reef regimes are most often characterised by their benthic components; however, complex dynamics are associated with losses and gains in both fish and benthic assemblages. To capture this complexity, we synthesised 3,345 surveys from Hawai‘i to define reef regimes in terms of both fish and benthic assemblages. Model-based clustering revealed five distinct regimes that varied ecologically, and were spatially heterogeneous by island, depth and exposure. We identified a regime characteristic of a degraded state with low coral cover and fish biomass, one that had low coral but high fish biomass, as well as three other regimes that varied significantly in their ecology but were previously considered a single coral dominated regime. Analyses of time series data reflected complex system dynamics, with multiple transitions among regimes that were a function of both local and global stressors. Coupling fish and benthic communities into reef regimes to capture complex dynamics holds promise for monitoring reef change and guiding ecosystem-based management of coral reefs.

## Introduction

With the unprecedented global decline of coral reefs in recent decades^1–4^, the paradigm of reefs as robust and predictable^5–7^ has given way to the recognition of reefs as dynamic complex systems^8,9^. As such, they display critical tipping-points, which if crossed can lead to abrupt changes in ecosystem structure and function, known as regime shifts^8,10–13^. Once manifested, these new regimes are often associated with some degree of stability due to strong reinforcing feedback mechanisms^14,15^. However, both theory and observation emphasize that a particular regime is not always a stable, unchanging condition. Rather, a particular regime can be characterized by dynamic fluctuations of the system around a specific attractor^16,17^.

To date, regime shifts on coral reefs have largely been described based on changes in benthic community structure. The most vividly described example is a shift from coral to macroalgae dominance^4,18–21^. However, shifts to other benthic regimes have been proposed, such as reefs becoming dominated by corallimorpharians, soft corals, and sponges^22^. In the Hawaiian archipelago, Jouffray *et al*.^23^ examined the relative abundance of a variety of benthic organisms to describe the co-occurrence of three distinct reef regimes dominated by corals and calcifying algae, macroalgae, or turf algae. Similarly, large-scale studies across the Pacific highlight the variability in cover of coral, crustose coralline algae, and macroalgae regardless of human population status^24^, and that inhabited and uninhabited islands are better differentiated by the cumulative cover of fleshy versus calcifying benthic organisms^25^. Together, these studies highlight the value of defining reef regimes beyond the binary view of coral *versus* macroalgae.

Fish abundance and diversity play a critical role for regime shifts on coral reefs^4,13,21,22,26^, and management of fish biomass has been advocated as an important and tangible tool to steer reefs away from regime shifts^27,28^. However, the reciprocity between fish and benthic communities is strong, and cause and effect can be difficult to discern^14,29^. This leads to a “chicken-and-egg” situation where fish are either viewed as a driver of benthic reef regimes^23,30^, or considered as a response variable to changes in benthic communities^31,32^.

In this study we embrace this reciprocity by providing an integrated approach that examines fish *and* benthic functional groups as the defining elements of reef regimes. This definition of regimes is broader than that of an alternative stable state or an equilibrium state, and describes the configuration of fish and benthic functional groups inclusive of their dynamics^33^. Using a spatially high-resolution biological dataset for coral reefs in the main Hawaiian Islands we define multiple regimes, describe spatial variation in regimes, and examine ecological patterns across regimes in terms of species composition and diversity. We then investigate variation across time within sites to explore the diversity of transitional pathways among regimes to illuminate our understanding of reef dynamics.

## Methods

Existing data from underwater visual surveys of fish and benthic assemblages were collated from seven monitoring programs for the main Hawaiian Islands. Each dataset was transformed into a consistent format and checked for errors. Data were only included in the analysis if benthic and fish surveys were co-located at a unique latitude and longitude and were from forereef habitats at depths of 0 to 30 meters. We also tested for the sensitivity of results to including data from different depth zones between 0 and 30 meters (0–20 meters, and 5–20 meters), and found no effect (Supplementary Figure S1). The majority of surveys were between 10 and 20 meters, so these depth ranges were chosen as they allowed for testing sensitivity to inclusion of shallow depths (<5 meters) and deeper depths (>20 m). The majority of the data (98%) were from 2000–2013. A total of 3,345 unique sites, defined as a survey location with a unique latitude and longitude, were used in the analyses. To account for spatial autocorrelation, means were taken for surveys within a defined distance of 300 meters (Supplementary Figures S2 and S3), resulting in an overall sample size of 1,027. The mean was used for sites with data across multiple years. Data sources, survey methods, and other meta-data are provided in the Supplementary Material.

To account for differences in survey method, fish data were standardised using calibration factors to the NOAA Biogeography Program belt transect since that method had the greatest consistency with the majority of other programs^34^ (Supplementary Table S1). Calibration factors were developed using an automated software program that utilises general linear models and Monte Carlo simulations^35^. Calibrations were calculated by species where possible based on the following decision rules: (1) a minimum of 10 paired observations were available within an island, (2) observations were not dominated by zeros – if they were, a delta model was run in which occurrences were modeled separately from non-occurrences, (3) residuals were normally distributed – if not, data were log-transformed and the model was rerun and checked again. If a species did not pass this series of rules, a calibration factor for each combination of family and trophic level (e.g. zooplanktivorous wrasses) was calculated. If a calibration factor could not be calculated at the family and trophic level, then a global calibration for the entire method was used. For all subsequent analyses, density estimates were based on calibrated densities of raw data. Benthic surveys were not calibrated as previous results found no large bias associated with percent cover among the methods used^36^ (Supplementary Table S1).

The biomass of individual fishes was estimated using the allometric length-weight conversion: *W* = *a*TL^*b*^, where parameters *a* and *b* are species-specific constants, TL is total length (cm), and *W* is weight (g). Length-weight fitting parameters were obtained from a comprehensive assessment of Hawai‘i specific parameters (Donovan *et al*., unpublished data) and FishBase^37^. Several fish species were removed from fish biomass calculations if aspects of their life history led to inaccurate counts with visual surveys, such as cryptic benthic species, nocturnal species, and pelagic schooling species. Likewise, manta rays were excluded, as their size is difficult to visually estimate and they have high biomass but are encountered infrequently. Additional methodology was developed for dealing with outliers in the fish data, accounting for extreme observations of schooling species. Extreme observations in the database were defined by calculating the upper 99.9% of all individual observations (e.g. one species, size and count on an individual transect), resulting in 26 observations out of over 0.5 million, comprised of 11 species. The distribution of individual counts in the entire database for those 11 species was then used to identify observations that fell above the 99.0% quantile of counts for each species individually. These observations were adjusted to the 99.0% quantile for analysis.

Fish and benthic assemblages were analyzed primarily at the level of functional groups. Benthic assemblages were broken into major functional groups including coral, macroalgae, turf algae, crustose-coralline algae, and other benthic cover (e.g. sponges, sand, basalt rock, recently dead coral). Other benthic cover was not broken down further due to limitations from incorporating data from different methods with different definitions for other benthic taxa. The fish assemblage was characterised into three trophic groups; herbivores, secondary consumers, and predators. Herbivores were further subdivided based on their feeding mode into browsers, grazers and scrapers following Edwards *et al*.^38^, which have been suggested as important indicators of resilience on coral reefs (Supplementary Table S2)^4,29,39,40^. Browsers were defined as those herbivores that feed on macroalgae and associated epiphytic material and are important for reducing cover of competing macroalgae. Grazers are considered those fishes that feed largely on algal turfs, which can limit the establishment of macroalgae, and scrapers also feed on algal turfs but can remove the reef substratum, opening space for coral recruitment^41,42^. Secondary consumers included corallivores, omnivores, invertivores, and planktivores. These groups were not further subdivided because they tend to have unstable biomass estimates (e.g., planktivores usually occur in large numbers with patchy distributions) so are not estimated well with transects, and thus may provide spurious results when considered independently. Predators were defined as large piscivorous species, such as sharks, jacks, and barracuda (Supplementary Table S2)^43^. Additional functional groups that have been shown to relate to reef resilience, such as urchins and *Acanthaster* spp., could not be included because data were not available.

Before analysis, all data were fourth root transformed and centered to meet the assumptions of linear models and all variables were standardised to the same scale. The fourth root transformation was chosen because it was strong enough to meet assumptions for all variables, such that a common transformation could be used for all 10 variables.

Regimes were identified using model-based clustering with the *mclust* package in R^44^ with the 10 fish and benthic functional groups as inputs. The cluster analysis is based on a probability model where each cluster is a mixture of multivariate normal distributions composed of the densities of each component, and each observation is assigned to a cluster based on the probability of membership given the observation. The *mclust* function uses three strategies for defining clusters: 1) initialization of the model with model-based hierarchical clustering, 2) maximum likelihood estimation with the expectation-maximization algorithm, and 3) model selection and the number of clusters that are approximated with Bayes factors and Bayesian Information Criterion^45^ (Supplementary Figure S4). Uncertainty in a point’s assignment to a regime was obtained during the clustering process by subtracting the probability of the most likely regime for each observation from one^44^.

The 10 multivariate benthic and fish functional groups were visualised with a non-metric multidimensional scaling plot using the *metaMDS* function in the *vegan* package in R^46^. A Bray-Curtis distance matrix was created with 2-dimensions and a maximum of 50 random starts to search for a stable solution and avoid getting trapped in a local optima^47^. Multivariate dispersion was also calculated for each regime and tested with an analysis of multivariate homogeneity of group dispersions with the *betadispr* function in the *vegan* package.

Coral species richness was examined across regimes with an Analysis of Variance, and contrasts and confidence intervals were calculated with Tukey’s honest significant differences where coral richness was the response variable and regimes were the explanatory variable. The community composition of corals was also examined by calculating the proportional cover of the four most abundant species within each regime, including *Porites lobata*, *Pocillopora meandrina*, *Porites compressa*, *Montipora capitata*. Coral species were also classified with a trait-based approach into competitive, stress-tolerant, generalist or weedy species following Darling *et al*.^48^, with additional species specific information on bleaching tolerance, and were compared across regimes.

In the Hawaiian Islands, seascape variables such as depth, habitat complexity, and wave exposure have been shown to be important predictors of fish assemblages^49–52^. As a result, spatial patterns across regimes were examined by comparing the proportion of each regime at each island, and by comparing the proportion of sites within a regime that were located on north, south, east, and west facing shores across islands. Additionally, depth and habitat complexity, measured as the maximum rate of change in seafloor slope (i.e. slope of slope), were calculated for each point from LiDAR derived bathymetry within a 60 m radius of each survey location^53^, and were compared across regimes with an Analysis of Variance and post-hoc Tukey multiple comparisons with either depth or complexity as response variables and regimes as explanatory variables.

Temporal transitions between regimes were assessed by predicting the regime for each year at each site individually with the function *predict.Mclust* in R. Predictions were retained if there was at least a 95% probability of the regime prediction belonging to that regime, and only sites where predictions were available for at least three years between 2000 and 2016 were retained. We used an extended dataset for the transition analysis encompassing a greater number of years compared to the dataset used to characterise the regimes, which ended in 2013. The extended dataset was not used previously in characterizing the regimes in order to avoid confounding effects of coral bleaching events that occurred in 2014 and 2015, as widespread bleaching was unobserved in all previous study years. A total of 80 sites were included in the temporal data set, and patterns of regime transitions were compared by calculating the frequency of a given transition as a proportion of the total number of possible transitions (n = 261). We also tested the sensitivity of analysing data from all 80 sites compared with only analysing those with longer time series (>4 or 6 years) by calculating binomial confidence intervals for each transition in each case. These sites also tended to represent permanent monitoring stations, which allowed for testing for the sensitivity to using observations from locations that may shift spatially from year to year. Binomial confidence intervals for each transition in each case were produced with the *binconf* function in the *Hmisc* package in R^54^ using the Wilson interval.

Finally, we tested the hypothesis that local and global human impacts will result in some transitions among regimes being more likely than others. Each observed transition was treated as a replicate, and the geographical position was used to obtain a value for a local impact represented as human population density within a 15 km radius, and a global impact represented as the degree heating weeks at the height of the 2015 bleaching event (Supplementary Material). The probability of a given transition was estimated with a Bayesian binomial model for each variable where there were at least four occurrences for a given transition as a function of either human population density or degree heating weeks. More details of the Bayesian binomial model are in the Supplementary material.

All analyses were conducted in the R environment for statistical computing version 3.3.0^55^.

## Results

### Identifying reef regimes

**Figure 1:**
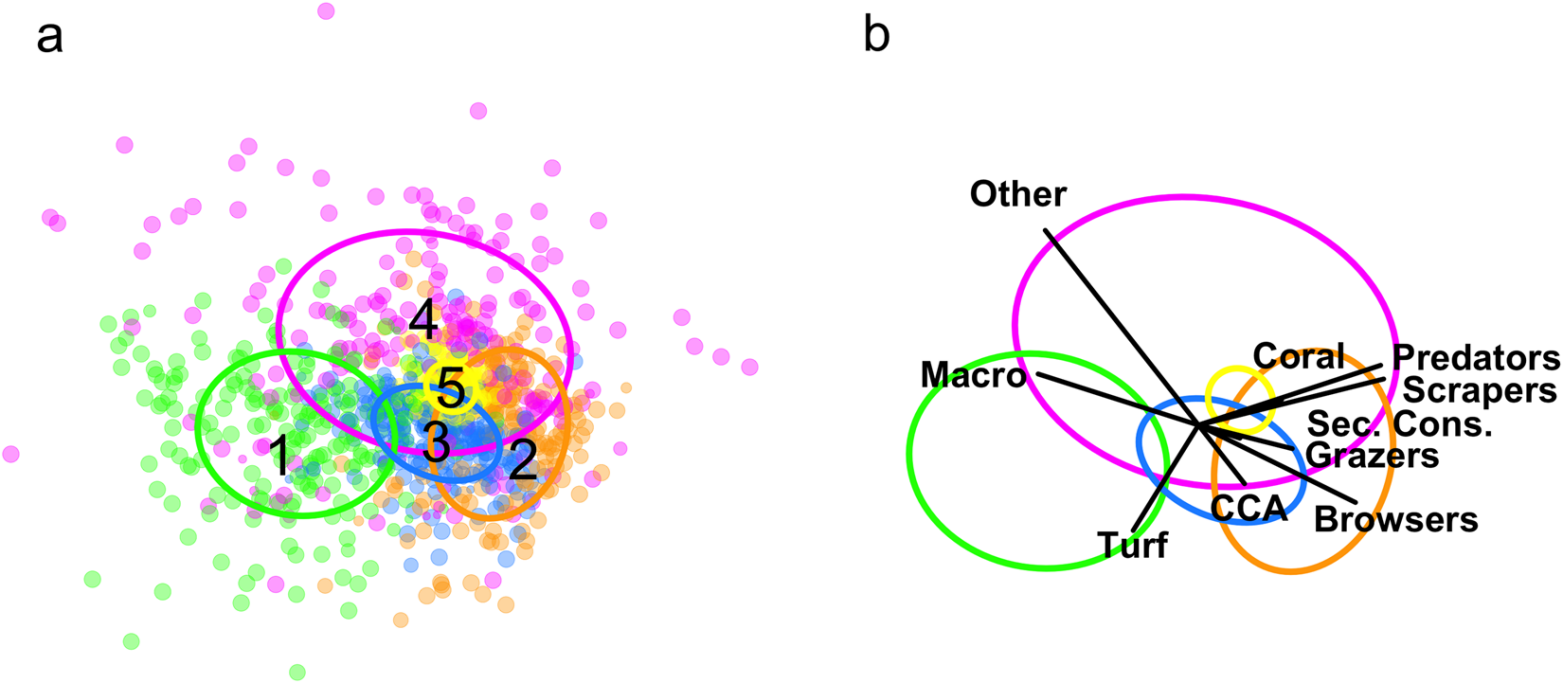
Non-metric multidimensional scaling plot of all data used in analysis of reef regimes (stress = 0.20). The size of points corresponds to uncertainty probabilities and are coloured by regime as classified by model-based clustering, with ellipses drawn around 50% of the data within each regime and labeled with numbers that correspond to discussion throughout the text (**a**), and ellipses with vectors corresponding to variables used in the analysis (**b**). Note, nMDS plot is a visual representation of the multi-dimensional data, but did not contribute to defining regimes, which was accomplished independently using model-based clustering. CCA is coralline algae, and Other is other benthic cover.

Our results revealed five distinct regimes based on model-based cluster analysis of 10 variables, representing fish and benthic functional groups, from a total of 3,345 reef surveys across the Hawaiian archipelago (based on final model selection, Supplementary Figure S4). Non-metric multidimensional scaling separated the variables in multivariate space (Fig. 1); with the biomass of each fish functional group inversely correlated with either turf or macroalgal cover (Fig. 1b).

**Figure 2:**
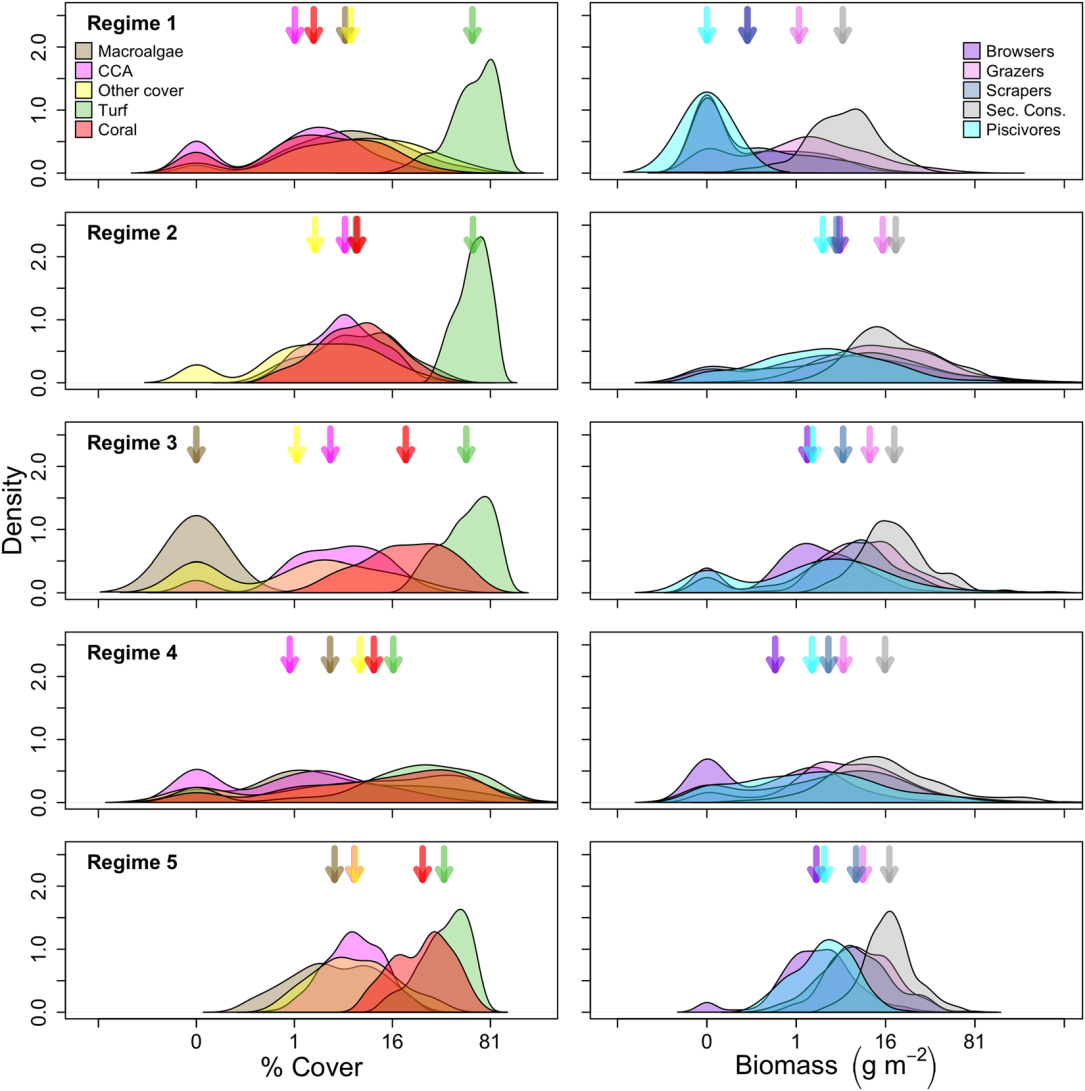
Kernel density of fish (right) and benthic (left) variables for each regime with arrows corresponding to the respective mean values from Table 1. Note, x-axes are fourth-root transformed to same scaling as used in cluster analysis.

The five identified regimes varied significantly in the composition of fish and benthic functional groups, reflecting differences in ecological structure and function (Table 1, Fig. 2). Regime 1 was characteristised with overall low coral cover and low fish biomass (especially predators), and a benthos characterised by turf algae, macroalgae, and other cover. Regime 2 had the highest fish biomass overall, with exceptionally high biomass of browsers, and a benthos dominated by turf algae. Coral cover in this regime was higher than in regime 1, but lower than all other regimes. Regime 3 had moderately high fish biomass across all functional groups, and high coral and turf cover and a complete absence of macroalgae. Regime 4 also had moderately high fish biomass across all functional groups, and a benthos with high coral cover and a mixture of macroalgae, turf algae, and other cover. Regime 5 had moderate fish biomass across all functional groups, in particular lower predator biomass when compared with regimes 3 and 4, and the highest mean coral cover across all regimes, high crustose coralline algae cover and comparatively low turf algae cover. Overall, multivariate variance (dispersion) differed across regimes (*p* < 0.01), with similar variability in regimes 1 and 2, regime 4 being the most variable, and regime 5 the least variable (Table 1).

**Table 1:** Summary of variables used to identify regimes, including mean and standard error by regime (in parentheses).

	REGIME (mean (SE))				
	1	2	3	4	5
Coral (%)	5.8 (0.5)	9.8 (0.5)	26 (1.4)	23.5 (1.6)	31.1 (1)
Macroalgae (%)	10.3 (0.9)	10.4 (0.6)	0 (0)	13 (1.5)	6.2 (0.4)
Other benthic cover (%)	13 (1)	5.4 (0.4)	5.5 (0.7)	22.6 (1.8)	9.6 (0.6)
Turf (%)	65.6 (1.4)	64.7 (0.9)	60.3 (1.6)	31.9 (1.9)	43 (1)
Coralline Algae (%)	3.5 (0.4)	7.2 (0.4)	6.5 (0.5)	5 (0.7)	8.1 (0.4)
Browsers (g m^−2^)	1 (0.2)	20.5 (3.4)	5.2 (1)	3.1 (0.7)	3.9 (0.3)
Grazers (g m^−2^)	5.1 (0.7)	25.4 (2.1)	16.5 (1.9)	12.2 (1.6)	11.7 (0.7)
Scrapers (g m^−2^)	1.1 (0.2)	15.1 (1.5)	11.8 (1.4)	12.6 (1.7)	10.6 (0.7)
Predators (g m^−2^)	0 (0)	9.7 (1.5)	8.7 (1.5)	8.3 (1.1)	4.1 (0.3)
Secondary Consumers (g m^−2^)	7.5 (0.5)	27 (1.8)	23.4 (1.5)	28.9 (3.8)	19.3 (0.7)
Total number of sites	205	250	158	200	214
Multivariate dispersion	1.702	1.847	1.675	2.479	1.082
Complexity (slope of slope)	6.5 (0.4)	12.1 (0.4)	10.7 (0.5)	10.3 (0.5)	13.5 (0.5)
Depth (meters)	8.8 (0.5)	11.4 (0.4)	8.3 (0.4)	10.2 (0.5)	9.6 (0.3)
North (% of total)	13.7	41.6	4.4	13.0	6.1
East (% of total)	23.9	22.0	3.8	21.5	21.0
South (% of total)	37.6	12.8	22.2	37.0	11.7
West (% of total)	24.9	23.6	69.6	28.5	61.2

Additional metrics used to compare regimes were total number of sites classified into each regime, multivariate dispersion based on an analysis of multivariate homogeneity of group dispersions, habitat complexity, depth, and proportion of sites within cardinal direction of coastlines.

**Figure 3:**
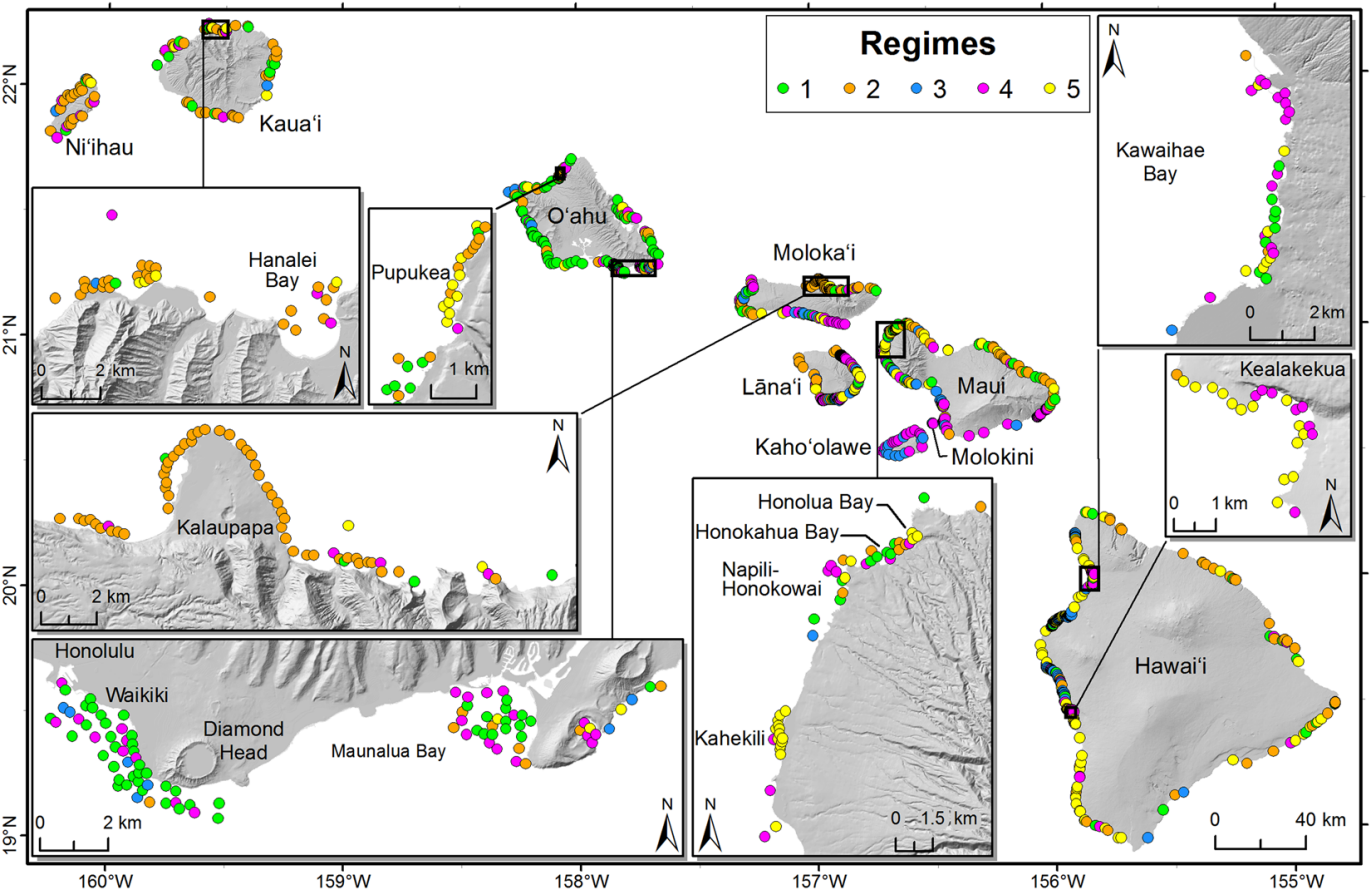
Spatial distribution of the 1027 sampling locations, coloured by regime, across forereefs of the main Hawaiian Islands. Map produced with ESRI ArcGIS Desktop 10.1 (http://desktop.arcgis.com/en/).

All regimes were present on all eight islands (Fig. 3), except for Kahoolawe, an uninhabited island inside a protected spatial management zone, which only consisted of regimes 3 and 4. The majority of sites on Niihau, a privately-owned island, which has a small human population (<200 residents) and limited public access, were regime 2 (68%). More than half of the sites on Oahu, the most populated island with over 1 million residents, were regime 1 (55%), and Hawai‘i Island, the largest island, was dominated by regime 5 (39%) (Table 1). We also found regimes were distributed differently according to the dominant cardinal direction of the coastline (north, south, east, west). Regime 1 and 4 were predominantly distributed across east (24% and 22%, respectively), south (38% and 37%), and west (25% and 29%) shores, regime 2 was found on north facing shores 42% of the time, while regimes 3 and 5 were both found on west facing (leeward) shores 70% and 61% of the time, respectively (Table 1). Further, two of the regimes correlated with depth and habitat complexity: regime 1 occurred in shallow low complexity habitat (e.g., pavement) (Table 1; Tukey multiple comparison of means: *p* ≤ 0.01), while regime 2 occurred in deep rugose habitat (e.g., large basalt boulders) (*p* < 0.01). Regime 5 also occurred in more complex habitats than the other two coral regimes (*p* < 0.01). There were no significant differences in depth or complexity for the other regimes (Table 1).

To understand how ecological attributes varied across regimes, we investigated patterns of species composition of corals, and richness for corals. There was no significant difference in the species richness of corals across regimes (F_4,142_ = 1.6, *p* = 0.16); however, the composition of the four most common coral species was variable. *Porites lobata*, a stress tolerant species, occurred frequently across all regimes, but often dominated in regimes 3 and 5. *Pocillopora meandrina*, a competitive species, occurred most frequently in regime 2, while *Porites compressa*, a weedy species, occurred most frequently in regimes 4 and 5, and *Montipora capitata*, also a competitively dominant species, occurred in relatively low frequencies across all regimes.

### Assessing regime shifts through time

**Figure 4:**
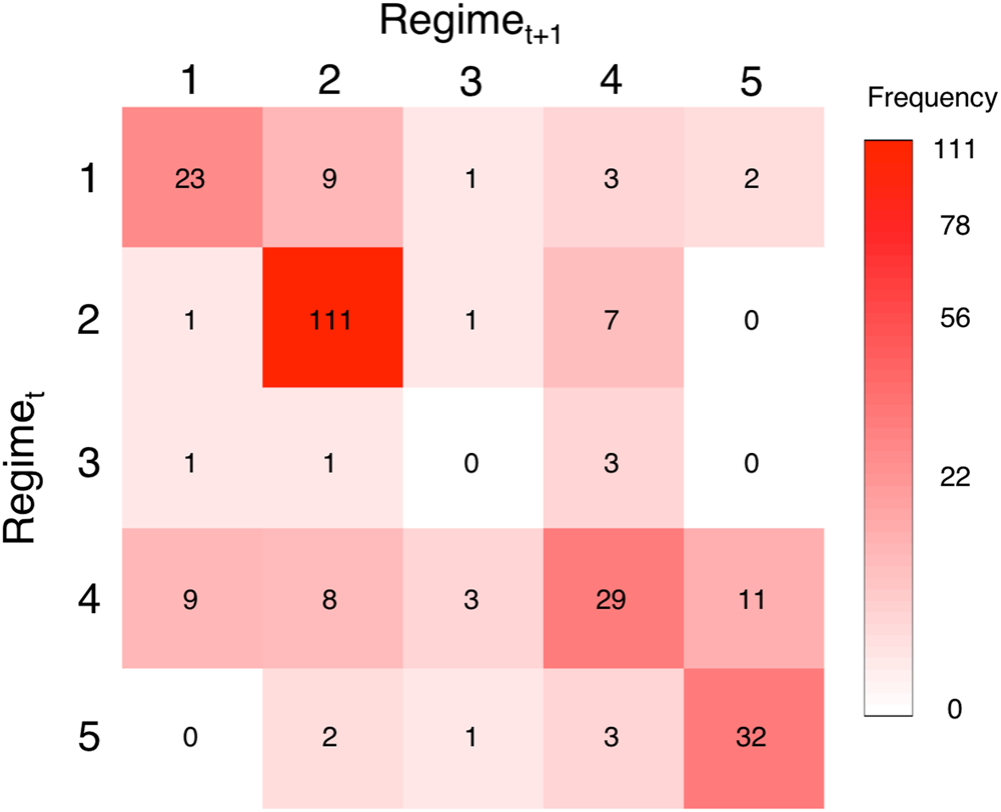
Summary of transitions between regimes over time with darker red corresponding to greater frequency of observation from 80 sites and 261 observed transitions. Numbers in each cell are the total number of transitions observed for that combination of regime before a transition (Regime_*t*_) and after a transition (Regime_*t*+1_) for a given site, thus the diagonal are those that remained the same.

A total of 261 regime transitions among years across 80 sites were observed in the dataset, and we analyzed the frequency of occurrence for a given transition for each combination of regimes (Fig. 4, Supplementary Figure S5). Regime 1 most often remained unchanged (61%), and otherwise switched to regime 2 (24%), or to a lesser extent to regime 4 (8%) or regime 5 (5%). We observed regime 2 repeatedly remaining the same (93%), making this pattern the most frequently observed overall. Regime 3, while not well represented in the time series, never remained the same, and otherwise switched to regime 2 or regime 4. Regime 4 remained the same half of the time (48%), and otherwise switched to every other regime. Regime 5 also frequently remained the same (83%) and was not observed transitioning directly to regime 1. Results were similar when these patterns were compared to calculations based on the subset of sites from permanent monitoring stations with at least four or six years of data, with common patterns observed in all cases (Supplementary Figure S6).

We also plotted the observations in multivariate space for several sites with longer time series that represent different areas spatially (Supplementary Figure S7). Honolua Bay, located in northwest Maui, showed a trajectory of decline in coral cover, starting in regime 5 and ending in regime 1, over the 12-year period. Conversely, two nearby embayments instead remained the same regimes; Honokahua Bay remained in regime 1 across the four years of data, and Napili-Honokowai remained in regime 4. Kahekili, also on Maui, switched to regime 1 after two years in regime 4 and then back to regime 5. Kalaupapa on the north coast of Molokai remained in regime 2, and observations were highly concordant across years. Molokini, a small islet off the south coast of Maui, remained in regime 4 across eight years of data, but sites were not tightly clustered, revealing more variation compared to Kalaupapa. Pupukea on the north shore of Oahu also transitioned from regime 1 to regime 2.

**Figure 5:**
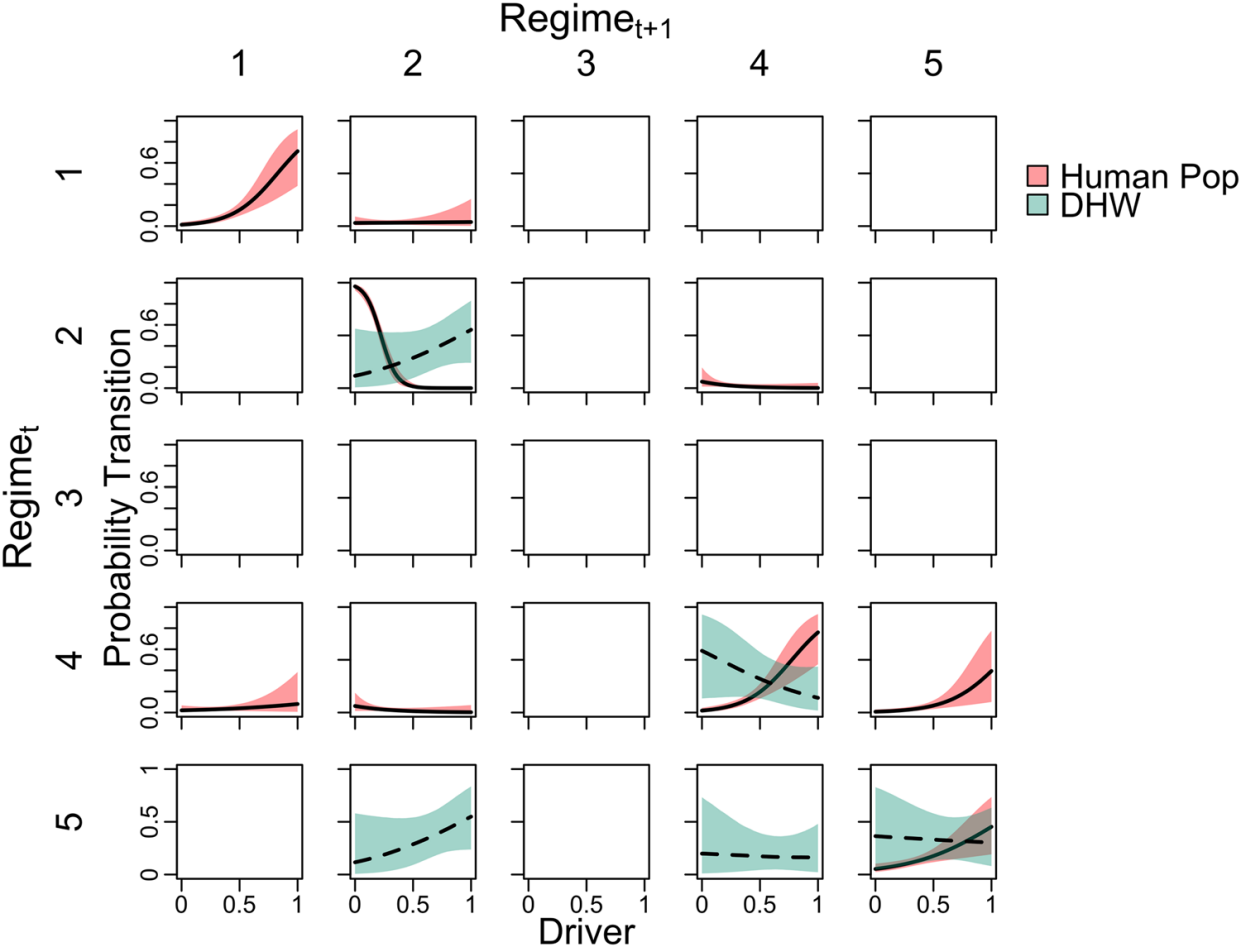
Analysis of probability of transitions among regimes given a local driver (human population density) and a global driver (extreme temperature event). Each panel represents a given regime from one time step (Regime_*t*_) to another (Regime_*t*+1_). The diagonals are those that remained the same from one time step to the next. Lines are Bayesian binomial models for human population density (solid, red) and degree heating weeks (DHW) (dotted, green) with 95% credible intervals. Values of both drivers are scaled between zero and one so they could be plotted on the same axis. Blank panels did not have enough data to estimate a relationship.

Certain transitions among regimes were significantly related to both local and global stressors (Fig. 5). Human population density was likely to have influenced several transitions among regimes, including regime 4 transitioning to regime 5. In contrast, regime 2 not changing was negatively related to human population, meaning there was a high probability of regime 2 transitioning to another regime at low levels of human population. Regimes 4 and 5 remaining the same, and regime 5 transitioning to regime 4, were also negatively associated with degree heating weeks at the height of the 2015 thermal stress event. In contrast, regime 2 remaining the same, and regime 5 transitioning to regime 2, were both positively associated with degree heating weeks.

## Discussion

We have demonstrated that by integrating fish and benthic functional groups, multiple reef regimes may exist, and pathways of change over time among regimes can be complex. We found a reef regime that was characteristic of a degraded reef with low fish biomass, low coral and high algal cover, but also identified a regime that has received less attention in the literature, which has low coral but high fish biomass. Further, we found evidence suggesting that what was previously considered one coral-dominated regime could potentially be divided into three distinct regimes that vary in their ecological composition. We also found particular sites followed trajectories of regime transitions that linked to known stressors of reefs (Fig. 5). Taken as a whole, the complex matrix of possible regime transitions supports the concept that one ecological transition from coral to macroalgae is not sufficient to describe reef dynamics^18,56^. The results also provide new insights into possible routes for both decline and recovery dynamics on coral reefs in relation to both local and global stressors that might help effectively prioritise management of these systems.

Quantification of regimes based on multiple components of the community enabled an examination of complex reef transitions, which provide an important context for understanding responses to disturbance. For example, Honolua Bay in northwest Maui has followed a classic trajectory of decline from coral to algal dominance, largely as a result of sedimentation^57^. However, the trajectory was not a single transition, as it began as regime 5 (high coral and high fish biomass) and transitioned to regime 4 and then regime 2 before ending in regime 1 (degraded) (Supplementary Figure S7). This multi-transitional trajectory provides an example of how the progression between regimes could be used to monitor subtle changes before a reef transitions to a degraded regime. Other time series in our dataset contradicted the expectation that degraded reefs are hard to reverse^58^, and exhibited patterns of recovery in coral cover and fish biomass, by transitioning from regime 1 (low coral and fish biomass) to regimes 2, 4 and 5 (Fig. 4). For example, Kahekili is a marine managed area where fishing of herbivores was restricted in 2009, and changes in both fish and benthic assemblages occurred following this closure^59^. During this time, Kahekili transitioned from regime 4 to regime 1 and recovered to regime 5 after fish biomass increased (Supplementary Figure S7), providing evidence of concurrent changes in the benthos following a specific fish harvest regulation, further supporting other studies that conclude that fisheries management can support reef resilience^60–62^. At several other sites coral reef regimes remained unchanged across time, particularly regime 2, bringing to question the common ecological mechanisms and management approaches that result in these patterns.

Species composition varied among regimes, with potential implications for how different regimes may respond to future disturbance events. Coral composition followed a pattern described by Jokiel *et al*.^63^, where *Pocillopora meandrina* dominates high wave energy communities (e.g., regime 2 on north facing shores), *Porites compressa* reefs occur in calmer environments (e.g., regimes 4, 5), and *Porites lobata* reefs occur in moderate environments (e.g., regime 3). Importantly, these coral species also have different tolerances to extreme heating events, both in terms of their susceptibility to bleaching mortality and their ability to recruit and regrow post-disturbance^48,64^. This is especially important given that in 2014 and 2015, the Hawaiian Archipelago suffered extreme coral bleaching with an estimated 20–50% coral mortality in some areas^65–67^. We found that certain transitions among regimes were both positively and negatively associated with thermal stress by testing whether certain transitions were more likely before and after the event in 2015 (Fig. 5). The probability of regime 4 and 5 remaining the same was lower with increasing thermal stress, which meets expectations given relatively higher overall coral cover, and higher cover of thermally sensitive species (e.g., *Porites compressa*) in those regimes (Table 1, Fig. 2). Interestingly, the probability that regime 5 (high coral, high fish) will transition to regime 2 (low coral, high fish) was also positively associated with thermal stress. This pattern may reflect that thermal stress directly reduces coral cover, with a possible lag-effect on fish assemblages^68^.

Our identification of five regimes is context dependent, and contingent on the choice and number of variables included in the analysis^69^. Future research is needed to better understand boundaries that may exist between these regimes and their sensitivity to including other reef components, such as urchins or microbes. We also acknowledge that the transient behaviour of some of the identified regimes could imply that these are merely temporary states between more stable regimes. However, in many instances regimes are dynamic and their behaviour can appear to be quasi-stable and transient - as opposed to stable - when observed over shorter time scales^16^. The challenge of discerning whether these are stable regimes or true transient states requires longer time-series. Furthermore, experimental approaches could test how ecological feedback mechanisms differ among regimes and may be related to reefs that undergo multiple transitions^13,14,70^. For example, regime 2 had high fish biomass, but not necessarily high coral cover and was often located along rugged, exposed coastlines (Figs 2 and 3). Looking at regime transitions as a function of human population density (Fig. 5), our study showed mixed results; transitions were both more and less likely to occur with increases in human population density. The rationale for these contrasting patterns remains to be explored, but could be due to the multitude of impacts associated with human population density as a proxy^71^. As this knowledge is built, using indicators of regimes and regime transitions can be incorporated into monitoring and management evaluation.

Understanding the spatial distribution of the regimes and their possible transitions provides crucial information for reef conservation, particularly for understanding and prioritizing management actions. For example, if the management goal is to maintain coral cover, it is important to consider that reefs can occur along a spectrum of coral cover, and that a low coral cover reef such as regime 2 is not necessarily a negative outcome of human pressures, but could instead reflect natural bounds set by the surrounding abiotic environment^24,72,73^. Also, management efforts may be successful when focused on regime 4 given the greater frequency of transitions observed for this regime, and the greater variance in composition without a difference in diversity, which have previously been shown to indicate a lack of resilience^74,75^. Finally, regime 1 is characteristic of a degraded state with high algae, low fish biomass, and low complexity, so management actions would need to focus on restoration in these locations. However, restoration may be costly, unrealistic within short time scales, and difficult to achieve over large areas^76^, as multiple feedbacks can make recovery difficult^77,78^.

By highlighting nuances in both the composition of regimes and transitions among regimes, we identify new insights into the ecological complexity of coral reefs. Hawai‘i provided a useful case study site by allowing us to address the need for descriptive reef regimes that capture the dynamics of both fish and benthic components of coral reefs in a data rich area. The results of the work are a step towards identifying key stressors, thresholds, and indicators of tipping points in Hawai‘i and provide a pathway for addressing pressing problems facing coral reefs worldwide. Future work is needed to tease apart the ecological mechanisms that underpin the different regimes, and to investigate how human and natural drivers determine their structure and function.

## Electronic supplementary material

Supplemental material

## Data Availability

Data are available in the Dryad data repository: https://doi.org/10.5061/dryad.rj083bv, and R scripts and data utilised for this study are archived at Donovan (2018)^79^.
