# Supplementary material for: Combining fish and benthic communities into multiple regimes reveals complex reef dynamics
Source: Sci Rep. 2018 Nov 16;8:16943. doi: 10.1038/s41598-018-35057-4 (PMC6240066; doi:10.1038/s41598-018-35057-4)
Supplement: Supplementary file 1 — Supplemental material [file 41598_2018_35057_MOESM1_ESM.pdf]

Supplementary Materials

Combining fish and benthic communities into multiple regimes reveals complex reef dynamics

Mary K. Donovan*, Alan M. Friedlander, Joey Lecky, Jean-Baptiste Jouffray, Gareth J. Williams, Lisa M. Wedding, Larry B. Crowder, Ashley L. Erickson, Nick A.J. Graham, Jamison M. Gove, Carrie V. Kappel, Kendra Karr, John N. Kittinger, Albert V. Norström, Magnus Nyström, Kirsten L.L. Oleson, Kostantinos A. Stamoulis, Crow White, Ivor D. Williams, Kimberly A. Selkoe

*Corresponding author: donovan.maryk@gmail.com

Spatial scale analysis

Before conducting analyses of reef regimes, semivariance and lacunarity analyses were used to assess the appropriate spatial scale to conduct the analysis. The goal was both to avoid violating assumptions of statistical independence, and to optimize our ability to detect patterns in community level data [1].

Semivariance

We analysed semivariance to determine the spatial scale at which spatial dependence in the raw data could be detected [2]. In geostatisical analysis, spatial variance, which follows the theoretical form:

$$\gamma(h) = \frac{1}{2n(h)}\sum_{i=1}^{n}[x_i - z(x_i + h)]^2$$

where x_i is the value of the observed variable at observation *i* and $n(h)$ is the number of observations located within h distance for each other, is plotted against lag distance h to construct a spatial variogram [3]. The empirical variogram can be constructed from the sampled data and compared to the theoretical form to estimate the distance at which spatial structure is detected. The distance where the semivariance asymptotes is referred to as the 'sill' and the corresponding value of h represents the 'practical range' where the variable of interest is no longer spatially autocorrelated [3].

For each variable used in the analysis, empirical semivariograms were constructed with the *variog* function in the *geoR* package in R [4] with 25 meter lag distances and a maximum distance of 1000 meters. This lag distance was chosen as the common length of the individual transects used in the analysis, and the maximum distance was used because the focus of the analysis was on observations that are close together and since spatial variance is greatest at short distances. Semivariance assumes that data are stationary, with no apparent spatial gradient, and are normally distributed. To meet this assumption of normality all variables were fourth-root transformed. Distances were calculated by converting geographic coordinates to *xy* coordinates based on an Albers Equal Area projection customized for the Hawaiian Archipelago with a central meridian of 167º and standard parallels of 26.86 minus the latitude and 20.5 minus the longitude where the latitude origin was set to 20º. Theoretical semivariograms were estimated with the *variofit* function in the *geoR* package, and assumed a spherical model with the following form:

$$\gamma(h) = 1 - 1.5\left(\frac{h}{\phi}\right) + 0.5\left(\frac{h}{\phi}\right)^3$$

where ϕ is the range parameter, and is only defined when $h > \phi$. Other bounded theoretical forms were also fit and spherical model was retained based on visual inspection of the fit. From the fitted variogram the practical range was estimated as the distance where semivariance was equal to 95% of the sill [5].

For all fish variables no levelling off of semivariance was found, and therefore no spatial dependence was observed. Benthic variables had a range of estimated spatial dependence ranging from 83 to 242 meters (Figure S2).

Lacunarity

To further determine the appropriate spatial scale to investigate reef regimes, we also considered how the distribution of spatial patterning in the data changed with spatial scale. Lacunarity is a scale dependent estimate describing the distribution of deviation from translational invariance [6]. In other words, it is a measure of how variable the texture of a geometric object is, and can be used to describe structure in spatial data. Lacunarity was quantified as:

$$L(s) = 1 + \frac{var(\mu(s))}{mean(\mu(s)^2)}$$

where a sequence of mean values for each variable, $\mu(s)$, was calculated for each window size s, which ranged from 25 to 2000 by 10 meter bins.

Lacunarity was plotted against distance (meters) for each variable and the distance at which the first derivative of lacunarity crossed zero was inspected. If spatial pattern is evident, we expect that lacunarity would increase before levelling off at the scale that maximizes the ratio of mean and variance. Thus, we calculated the distance at which the first derivative crossed zero. For all variables except coral cover we did not observe an initial increase in lacunarity. For coral cover, lacunarity reached an asymptote at 140 meters (Figure S3).

Summarizing data by spatial grouping

Based on the maximum distance for which we detected spatial autocorrelation to be a concern we determined a minimum distance between transects (242 meters), and calculated means for groups of points. In ArcGIS we added the point layer to a topology with a spatial cluster tolerance of 70 meters. This uses a 70 meter by 70 meter moving window to snap point locations together, where the maximum distance a coordinate could move to the centroid of a cluster is √2 * 70m. In practice, this results in varying sizes of clusters: the maximum distance between any two points in a cluster was 300 meters, and the minimum distance between any two cluster centers was 130 meters.

Model-based cluster analysis model selection

To identify regimes, model-based cluster analysis using the *mclust* package in R was performed [7]. Variables were modeled as Gaussian mixture models, and parameters were found with the expectation-maximization algorithm. The corresponding densities are ellipsoidal and can take different shapes based on geometric features (shape, volume and orientation), which are also parameterized in *mclust* (Figure S4) [7,8]. Model-based cluster analysis of the 10 fish and benthic variables revealed five distinct regimes based on a model selection of models with varying shapes. The final model was ellipsoidal, with varying volume, shape, and orientation (Table 1, Figure 2; logL = 6450.6, n=1027, df=329, BIC=10610.7)

Time series details

Temporal patterns across regimes were assessed for a total of 80 sites that were located throughout the study area (Figure S5). These sites were located across 14 reef tracts, so some sites were spatially clustered because they occur in areas that tend to be monitored more frequently. Overall, 261 transitions were observed (Table S3). At each site for each year the regime was predicted from the observed values of the 10 functional groups, and observations were retained for only those with at least a 95% probability of occurring in the predicted regime. We also plotted the observations in multivariate space for 8 time series with at least four years of data between 2000 and 2013 (Figure S7).

We tested the sensitivity of analysing data from all 80 sites compared with only analysing those with longer time series (≥ 4 years) by calculating binomial confidence intervals for each transition in each case (Figure S6). Binomial confidence intervals were produced with the *binconf* function in the *Hmisc* package in R[9] using the Wilson interval. Overall patterns were similar with overlapping confidence intervals in all cases (Figure S6).

Finally, we tested the hypothesis that local and global human impacts will result in in some transitions among regimes being more likely than others. This was accomplished by treating each observed transition as a replicate, and using the geographical position to obtain a value for human population density within a 15km radius, and the degree heating weeks at the height of the 2015 bleaching event. Degree heating weeks represents cumulative thermal stress

for a given location [10], and was calculated for November 1, 2015 based on products from NOAA Coral Reef Watch (httpe://coralreefwatch.noaa.gov) 5km v3 product. For human population density, all observations were used, and a model was fit as long as there were greater than 4 occurrences for the transition combination (Figure 5). For degree heating weeks, only transitions before and to 2016 were used (n=21) to capture the coral bleaching event in 2015, and several additional sites were included that had a greater uncertainty than the larger dataset to optimize the number of replicates.

We calculated the probability of a given transition with a Bayesian model that was fit with flat priors, where the model was:

$$y_i \sim Bernoulli(p_i)$$
$$logit(p_i) = \beta_o + \beta_1 x_i$$
$$\beta_o \sim N(0{,}1000)$$
$$\beta_1 \sim N(0{,}1000)$$

The model was fit using JAGS via the *rjags* package in R [11], with 25,000 iterations, with 5000 for adaptation and 10,000 for burn-in, leaving a posterior sample size of 10,000. Model convergence was monitored by running 2 chains with different initial values and calculating Gelman–Rubin statistics [12]. We found good convergence for all models with Gelman-Rubin estimates of equal to or nearly equal to 1 (Table S4). Model fits were also assessed with posterior predictive checks, where observed values are compared to simulated values from the model (Table S4).

Table S1. Description of datasets used in analyses of reef regimes from multiple sources that were compiled into a standardized format from large scale monitoring programs in the main Hawaiian Islands. SPC is stationary point count method, and LPI is line-point-intercept method.

Dataset	Fish method	Benthic method	N	Year range	Depth range (m)
Coral Reef Assessment and Monitoring Program - University of Hawaii[1]					
	Belt	video	52	1999 - 2002	1.0 - 13.0
		photo-quad	46	2003 - 2012	1.0 - 13.0
NOAA Coral Reef Ecosystem Program[2]*					
	Belt	LPI or photo-quad	117	2005 - 2008	7.5 - 17.0
	SPC	photo-quad	708	2008 - 2013	0.0 - 30.0
Division of Aquatic Resources[3]					
	Belt	photo-quad	512	2004 - 2012	0.0 - 15.2
	WHAP Belt	photo-quad	122	2003 - 2013	1.5 - 15.0
Fisheries Ecology Research Lab - University of Hawaii[4]					
	Belt	quadrat or photo-quad	242	1993 - 2013	0.0 - 16.7
NOAA Fish Habitat Utilization Study[5]					
	Belt	quadrat	766	2002 - 2008	0.5 - 32.0
National Park Service[6]					
	Belt	photo-quad	174	2004 - 2013	11.0 - 21.4
The Nature Conservancy Hawaii Marine Program[7]					
	Belt	photo-quad	606	2009 - 2013	1.0 - 20.7
			3345	1993 - 2013	0.0 - 30.0

[1]Benthic surveys were 100-meter long fixed transects, 20 photo-quadrats taken at 0.5 meters above substrate. Fish surveys were 25 by 4 meters, lasting between 10-15 minutes. See [13–15] for details.

[2]Fish surveys consisted of two methods, a 25 meter long belt transect with fishes > 20 cm surveyed in a 5 meter swath and smaller fishes surveyed in a 2 meter swath, and a stationary point count in a cylinder with a 15 meter diameter. Benthic surveys were either photo-quadrats or line-point-intercept collected along the fish transects. For details see [16]. * Coral Reef Ecosystem Program; Pacific Islands Fisheries Science Center (2013). National Coral Reef Monitoring Program: Stratified Random surveys (StRS) of Reef Fish, including Benthic Estimate Data of the U.S. Pacific Reefs since 2007. NOAA National Centers for Environmental Information. Unpublished Dataset. https://inport.nmfs.noaa.gov/inport/item/24447

[3]Fish surveys were 25 meters long by 4 meters wide belt transects surveyed by a pair of divers, and benthic surveys were photo-quadrats taken along the same transect lines [14]. WHAP methods consisted of 25 meter transects with continuous photo-quadrats, see [17] for details.

[4]Fish surveys consisted of a 25 meter by 5 meter transect with the diver swimming at a constant speed and recording count and size of all fishes, and benthic surveys consisted of *in situ* quadrats deployed randomly along the fish transect, with intercepts within the quadrat identified. See [18] for details.

[5]Fish consisted of a 25 meter by 5 meter transect with the diver swimming at a constant speed and recording count and size of all fishes. Benthic surveys were done *in situ* using a 1 m^2 quadrat divided into 100 squares and benthic cover reported at random intercepts of the squares and deployed at random intercepts along the fish transect. See [19,20] for details.

[6]Fish surveys were the same as [5] and [6], and benthic surveys were the same as [1].
[7]Fish surveys consisted of paired divers swimming at a constant speed along a 25 meter by 5 meter transect and counting and sizing all fishes, similar to [19]. Benthic surveys consisted of 25 photo-quadrats taken along the fish transect at random intervals, and later analyzed for intercept of 30 random points per quadrat.

Table S2. Species broken into functional groups used in analysis of reef regimes

Predators

Antennarius commersoni
Aphareus furca
Aprion virescens
Aulostomus chinensis
Carangoides orthogrammus
Caranx ignobilis
Caranx lugubris
Caranx melampygus
Caranx sexfasciatus
Carcharhinus amblyrhynchos
Carcharhinus galapagensis
Cephalopholis argus
Epinephelus quernus
Fistularia commersonii
Oxycheilinus unifasciatus
Pseudocaranx cheilio
Saurida flamma
Saurida gracilis
Scomberoides lysan
Seriola dumerili
Seriola rivoliana
Sphyraena barracuda
Sphyraena helleri
Synodus binotatus
Synodus dermatogenys
Synodus ulae
Synodus variegatus
Triaenodon obesus
Tylosurus crocodilus

Browsers

Calotomus carolinus
Calotomus zonarchus
Kyphosus bigibbus
Kyphosus cinerascens
Kyphosus hawaiiensis
Kyphosus vaigiensis
Naso lituratus
Naso unicornis

Grazers

Acanthurus achilles
Acanthurus blochii
Acanthurus dussumieri
Acanthurus guttatus
Acanthurus leucopareius
Acanthurus lineatus
Acanthurus maculiceps
Acanthurus nigricans
Acanthurus nigrofuscus
Acanthurus nigroris
Acanthurus olivaceus
Acanthurus triostegus
Acanthurus xanthopterus
Zebrasoma flavescens
Zebrasoma veliferum

Scrapers

Chlorurus perspicillatus
Chlorurus spilurus
Scarus dubius
Scarus psittacus
Scarus rubroviolaceus

Secondary Consumers

Abudefduf abdominalis
Abudefduf sordidus
Abudefduf vaigiensis
Acanthurus thompsoni
Albula glossodonta
Aluterus scriptus
Amblycirrhitus bimacula
Anampses chrysocephalus
Anampses cuvier
Apogon erythrinus
Apogon kallopterus
Apogon maculiferus
Apolemichthys arcuatus
Arothron hispidus
Arothron meleagris
Asterropteryx semipunctatus
Atherinomorus insularum
Balistes polylepis
Blenniella gibbifrons
Bodianus albotaeniatus
Bothus mancus
Bothus pantherinus
Brotula multibarbata
Callionymus comptus
Cantherhines dumerilii
Cantherhines sandwichiensis
Cantherhines verecundus
Canthidermis maculatus
Canthigaster amboinensis
Canthigaster coronata
Canthigaster epilampra
Canthigaster jactator
Canthigaster solandri
Caracanthus typicus
Carangoides ferdau
Centropyge fisheri
Centropyge flavissima
Centropyge interrupta
Centropyge loriculus
Centropyge potteri
Chaetodon auriga
Chaetodon citrinellus
Chaetodon ephippium
Chaetodon fremblii
Chaetodon kleinii

Chaetodon lineolatus
Chaetodon lunula
Chaetodon lunulatus
Chaetodon miliaris
Chaetodon multicinctus
Chaetodon ornatissimus
Chaetodon quadrimaculatus
Chaetodon reticulatus
Chaetodon tinkeri
Chaetodon trifascialis
Chaetodon unimaculatus
Cheilio inermis
Chromis acares
Chromis agilis
Chromis hanui
Chromis leucura
Chromis ovalis
Chromis vanderbilti
Chromis verater
Cirrhilabrus jordani
Cirrhitops fasciatus
Cirrhitus pinnulatus
Cirripectes obscurus
Cirripectes vanderbilti
Coris ballieui
Coris flavovittata
Coris gaimard
Coris venusta
Coryphopterus duospilus
Ctenochaetus hawaiiensis
Ctenochaetus strigosus
Cymolutes lecluse
Cymolutes praetextatus
Dascyllus albisella
Dendrochirus barberi
Diodon holocanthus
Diodon hystrix
Doryrhamphus excisus
Echeneis naucrates
Enneapterygius atriceps
Entomacrodus marmoratus
Epibulus insidiator
Eviota epiphanes
Evistias acutirostris
Exallias brevis
Foa brachygramma
Forcipiger flavissimus
Forcipiger longirostris
Genicanthus personatus
Gnathanodon speciosus
Gnatholepis anjerensis
Gnatholepis caurensis
Gomphosus varius
Goniistius vittatus
Gunnellichthys curiosus
Halichoeres ornatissimus
Hemiramphus depauperatus
Hemitaurichthys polylepis
Hemitaurichthys thompsoni
Heniochus diphreutes
Heteropriacanthus cruentatus
Hippocampus fisheri
Hippocampus kuda
Iniistius aneitensis
Iniistius pavo
Iniistius umbrilatus
Istiblennius zebra
Kuhlia sandvicensis
Labroides phthirophagus
Lactoria fornasini
Lutjanus fulvus
Lutjanus kasmira
Macropharyngodon geoffroy
Malacanthus brevirostris
Melichthys niger
Melichthys vidua
Microcanthus strigatus
Monotaxis grandoculis
Mugil cephalus
Mulloidichthys flavolineatus
Mulloidichthys mimicus
Mulloidichthys pflugeri
Mulloidichthys vanicolensis
Myripristis amaena
Myripristis berndti
Myripristis chryseres
Myripristis kuntee
Myripristis vittata
Naso annulatus
Naso brevirostris
Naso caesius
Naso hexacanthus
Naso maculatus
Nemateleotris magnifica
Neomyxus leuciscus
Neoniphon aurolineatus
Neoniphon sammara
Novaculichthys taeniourus
Omobranchus rotundiceps
Oplegnathus fasciatus
Oplegnathus punctatus
Opua nephodes
Ostorhinchus maculiferus
Ostracion meleagris
Ostracion whitleyi
Oxycheilinus bimaculatus
Oxycirrhites typus
Parablennius thysanius
Paracirrhites arcatus
Paracirrhites forsteri
Parapercis schauinslandi
Parupeneus chrysonemus
Parupeneus cyclostomus
Parupeneus insularis
Parupeneus multifasciatus
Parupeneus pleurostigma
Parupeneus porphyreus
Pervagor aspricaudus
Pervagor spilosoma
Plagiotremus ewaensis
Plagiotremus goslinei
Platybelone argalus
Plectroglyphidodon imparipennis
Plectroglyphidodon johnstonianus
Plectroglyphidodon sindonis
Pleurosicya micheli
Priacanthus meeki
Priolepis aureoviridis
Priolepis eugenius
Pristiapogon kallopterus
Pristiapogon taeniopterus
Pristilepis oligolepis
Pseudanthias bicolor
Pseudanthias hawaiiensis
Pseudanthias thompsoni
Pseudocheilinus evanidus
Pseudocheilinus octotaenia
Pseudocheilinus tetrataenia
Pseudojuloides cerasinus
Psilogobius mainlandi
Ptereleotris heteroptera
Pterois sphex
Rhinecanthus aculeatus
Rhinecanthus rectangulus
Sargocentron diadema
Sargocentron ensifer
Sargocentron punctatissimum
Sargocentron spiniferum
Sargocentron tiere
Sargocentron xantherythrum
Scorpaenodes kelloggi
Scorpaenodes parvipinnis
Scorpaenopsis brevifrons
Scorpaenopsis cacopsis
Scorpaenopsis diabolus
Sebastapistes ballieui
Sebastapistes coniorta
Stegastes marginatus
Stethojulis balteata
Sufflamen bursa
Sufflamen fraenatus
Taenianotus triacanthus
Thalassoma ballieui
Thalassoma duperrey
Thalassoma lutescens
Thalassoma purpureum
Thalassoma quinquevittatum
Thalassoma trilobatum
Trimma taylori
Upeneus arge
Wetmorella albofasciata
Xanthichthys auromarginatus
Xanthichthys mento
Zanclus cornutus

Table S3. Predicted regime by year for 80 sites where data were available for at least three years.

Latitude	Longitude	1993	1999	2000	2002	2003	2004	2005	2006	2007	2008	2009	2010	2011	2012	2013	2014	2015	2016
19.37	-155.90									5				5			5		5
19.43	-155.92						2		3	4									
19.46	-155.93									5				5			5		
19.47	-155.92						5		5	5									
19.47	-155.92						4		4	4									
19.48	-155.93						3		4	4									
19.49	-155.95						2		4	4									
19.51	-155.95									5				5			5		
19.55	-155.96									5				5			3		
19.63	-155.99									5				5			5		
19.65	-156.02									5				5					2
19.70	-156.05									5				4			5		
19.84	-155.98									5				5			5		
19.95	-155.87									5							5	5	
20.07	-155.86									5				5			5		
20.63	-156.50			4												4	3	4	4
20.74	-156.88								4		4				1				
20.94	-156.69											4	5	5	5				
20.94	-156.69										5	4	1		5				
20.94	-156.69										5	4		1	2				
20.94	-156.69											4	5	5	5				
20.94	-156.69											4	1	5	5				
20.94	-156.69										4	4			3				
20.94	-156.69											4	1		2				
20.94	-156.69										4	4	5	5					
20.95	-156.69											4	5		5				
20.95	-156.70										4	4	5		5				

20.95	-156.69				4	4			5			
20.95	-156.69					4	5	5				
21.00	-156.67	1	4	4			4					
21.00	-156.67	4	4	4					4			
21.00	-156.67	4	4	4								
21.01	-156.67	1	1	1								
21.01	-156.66	1	1	1								
21.01	-156.65	1	1	1					1			
21.01	-156.65	1	1	1					1			
21.01	-156.64	5	5	5								
21.17	-156.92		2	2		4	4	2	4			2
21.17	-156.92			2	2		4	1	4	2	2	2
21.18	-156.95						1	2	2			
21.18	-157.00					4	2	2	2			
21.18	-156.95		1	2	2	2	2	2	2	2	2	2
21.19	-157.01		2	2	2	2	2	2		2	2	2
21.19	-157.02			2			2				2	
21.19	-157.02				2		2	2				
21.19	-156.95		2	2	2	2	2	2	2	2	2	2
21.19	-156.95		2	2	2	2	2	2	2	2	2	2
21.19	-156.95		2		2			2				
21.19	-156.99		2	2	2	2		2	2		2	2
21.20	-156.95		2		2		2					
21.20	-156.99		2	2	2		2		2		2	
21.20	-156.95		2	2	2		2		2	2	2	2
21.20	-156.99					2		2	2			
21.21	-156.98		2			2		2	2	2		2
21.21	-156.98			2	4	1	2	2	1		2	
21.21	-156.96		2	2		2	2		2			

21.21	-156.98								2	2	2	2	2	2	2	2	2	2
21.21	-156.97								2	2	2	2	2	2	2	2	2	
21.22	-156.97								2	2	2	2	2	2	2	2	2	2
21.27	-157.69			4			4			4								
21.44	-157.79			4											4	4		
21.63	-158.08					1			1	1								
21.63	-158.08					1			1	1								
21.63	-158.07					1			1	1								
21.64	-158.07					2			2	2								
21.65	-158.06			4		1			3	1								
21.65	-158.06								5				5		2			
21.65	-158.06					4			2				2					
21.66	-158.06					1			1	1			2					
21.66	-158.06					1			1	1			2					
21.67	-158.05					1			1	1								
22.21	-159.51	2	2	4			2	2										
22.21	-159.50		4				3	2										
22.22	-159.50	4	2				4	2										
22.22	-159.50	4	4					4										
22.22	-159.50	4	4				4											
22.23	-159.50	1	1				2											
20.94	-156.69										4		4	5				
20.94	-156.69										4	4		5		5		
20.95	-156.69										4			1	4	5		

Table S4. Diagnostics for Bayesian binomial models of transitions as a function of human population density and degree heating weeks. Gelman-Rubin statistic assesses model convergence across the 2-MCMC chains, where a value of 1 approximates convergence. Posterior predictive check compares observed values to simulated values from the model and represents the probability that the test statistic from the simulated data is more extreme than the observed data, thus values of 0 or 1 represent lack of fit.

Transition	Gelman-Rubin Statistic				Posterior Predictive Check			
	Human population		DHW		Human population		DHW	
	B0	B1	B0	B1	mean	sd	mean	sd
1 – 1	1.00	1.00			0.56	0.45		
1 – 2	1.00	1.00			0.59	0.49		
1 – 3								
1 – 4								
1 – 5								
2 – 1								
2 – 2	1.00	1.00	1.00	1.00	0.52	0.53	0.58	0.52
2 – 3								
2 – 4	1.00	1.00			0.60	0.44		
2 – 5								
3 – 1								
3 – 2								
3 – 3								
3 – 4								
3 – 5								
4 – 1	1.00	1.00			0.59	0.44		
4 – 2	1.00	1.00			0.60	0.49		
4 – 3								
4 – 4	1.00	1.00	1.00	1.01	0.54	0.46	0.60	0.51
4 – 5	1.00	1.00			0.58	0.52		
5 – 1								
5 – 2			1.00	1.00			0.58	0.53
5 – 3								
5 – 4			1.00	1.00			0.62	0.53
5 – 5	1.00	1.00	1.00	1.00	0.54	0.51	0.59	0.53

Figure S1. Sensitivity of regime classification to depth limits used. The distribution of values is shown for each functional group including benthic (left) and fish (right) for 3 different depth intervals: 0-30 m (black), 0-20 m (red), and 5-20 m (blue). All intervals are overlapping, thus reflecting no large differences as a result of using all depths (black).

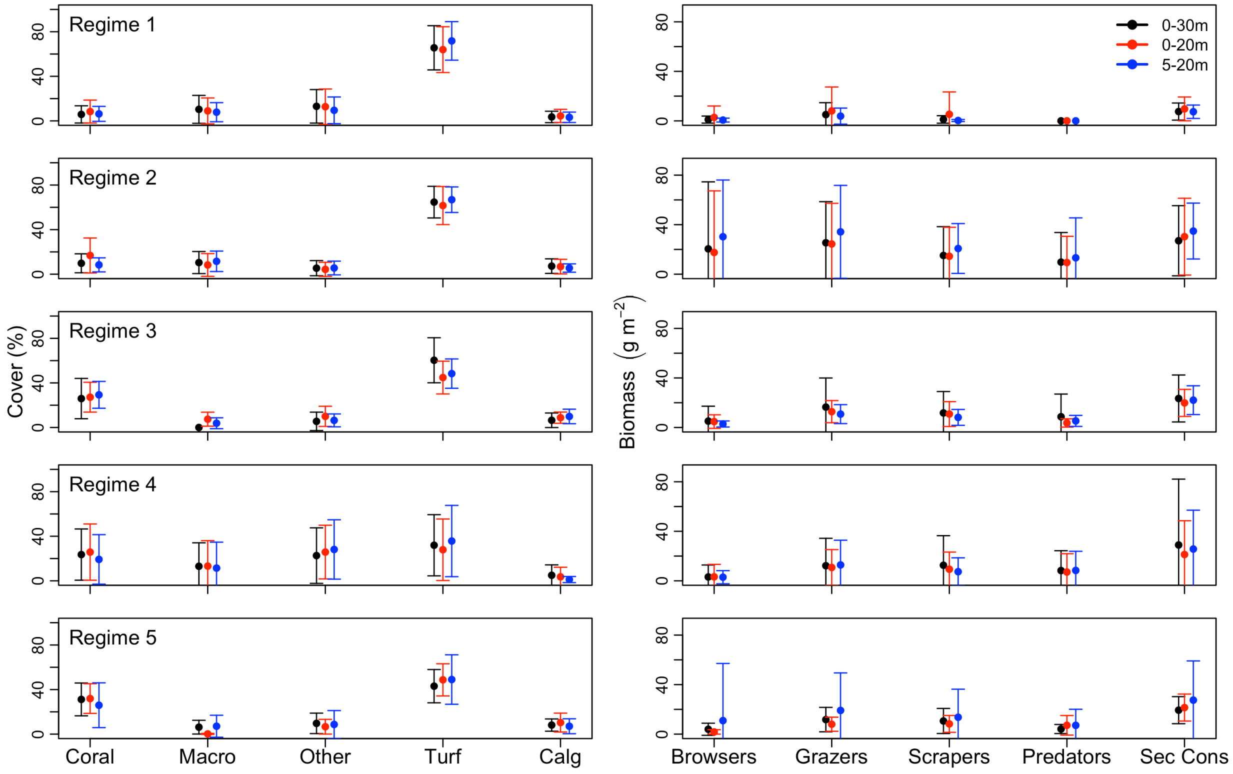

Figure S2. Semivariograms for benthic variables. Open circles and black lines are empirical semivariograms at 25 meter lag distances. Red lines are theoretical semivariograms based on a spherical model, and the vertical dashed lines corresponds to the estimated practical range in meters where the semivariance is 95% of the sill.

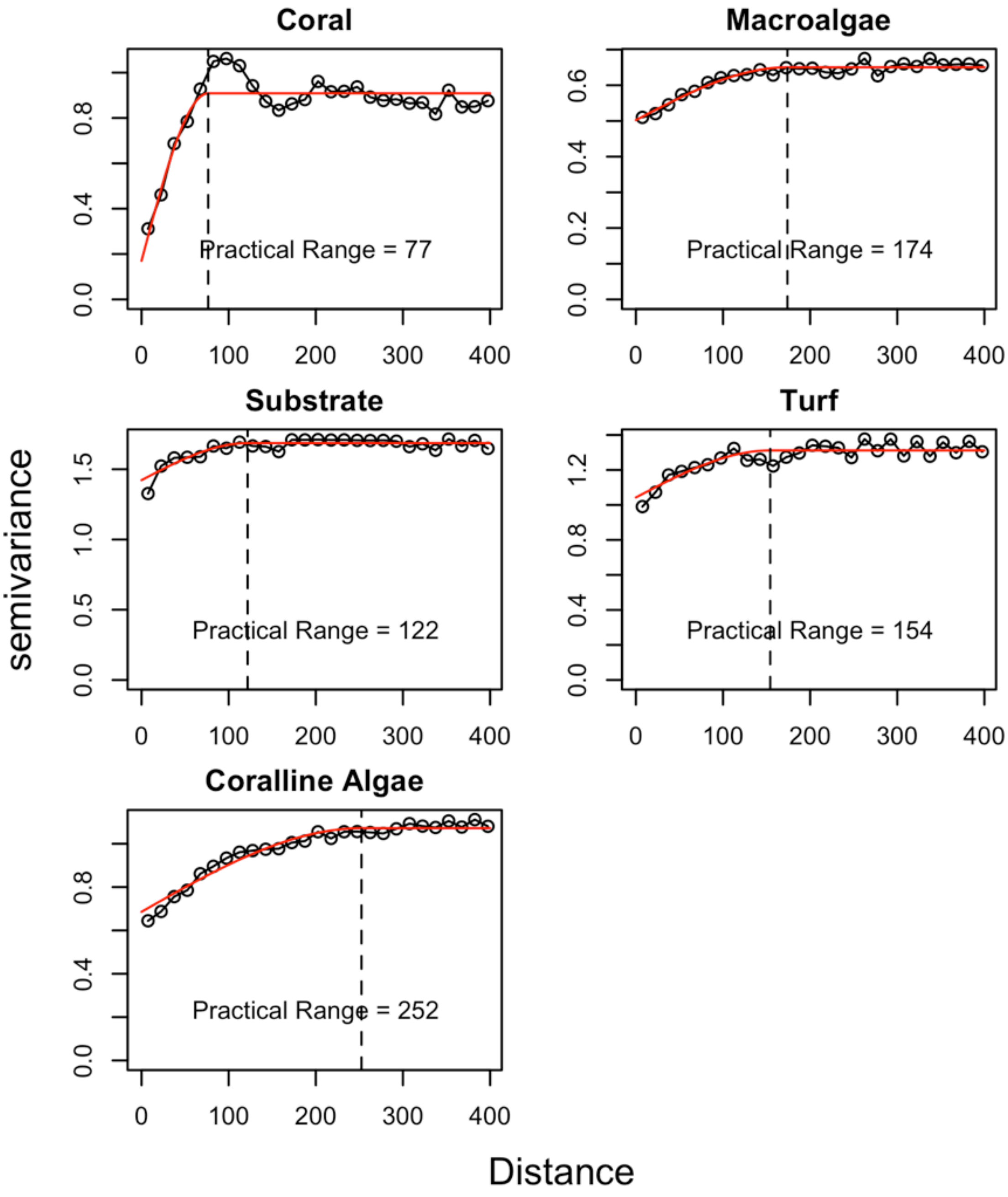

Figure S3. Lacunarity as a function of distance (meters) (A), and first derivative of lacunarity as a function of distance (B) for coral cover.

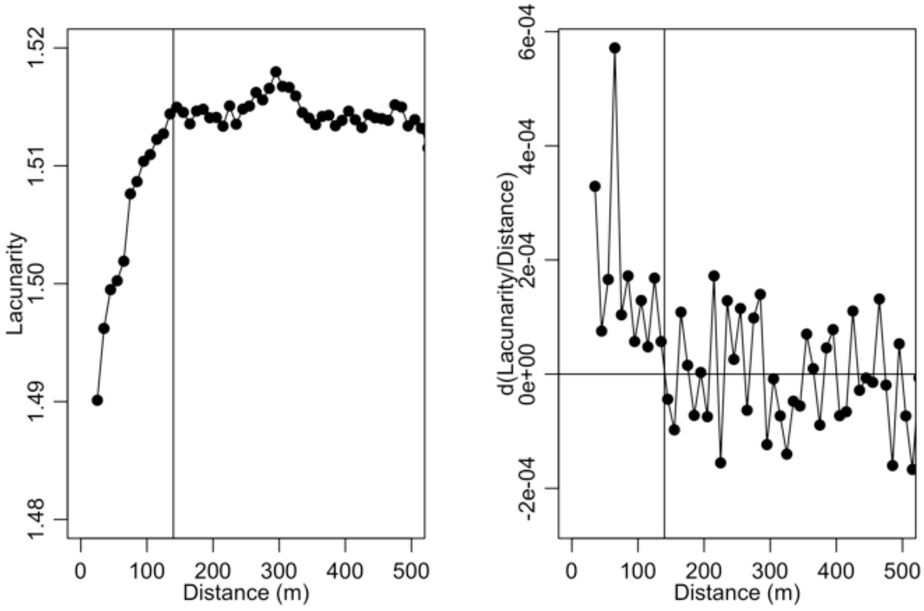

Figure S4. Plot of Bayesian Information Criterion (BIC) for cluster analysis model selection based on models with equal or variable geometric features and different numbers of clusters. The best model has the highest BIC (VVV, 5 components). Model parameterizations are: EII – equal volume, equal shape, VII – variable volume, equal shape, EEI – equal volume, equal shape, coordinate axes orientation, VEI – variable volume, equal shape, coordinate axes orientation, EVI – equal volume, variable shape, coordinate axes orientation, VVI – variable volume, variable shape, equal axes orientation, EEE – equal volume, equal shape, equal orientation, EEV – equal volume, equal shape, variable orientation, VEV – variable shape, equal volume, variable orientation, and VVV – variable volume, variable shape, variable orientation.

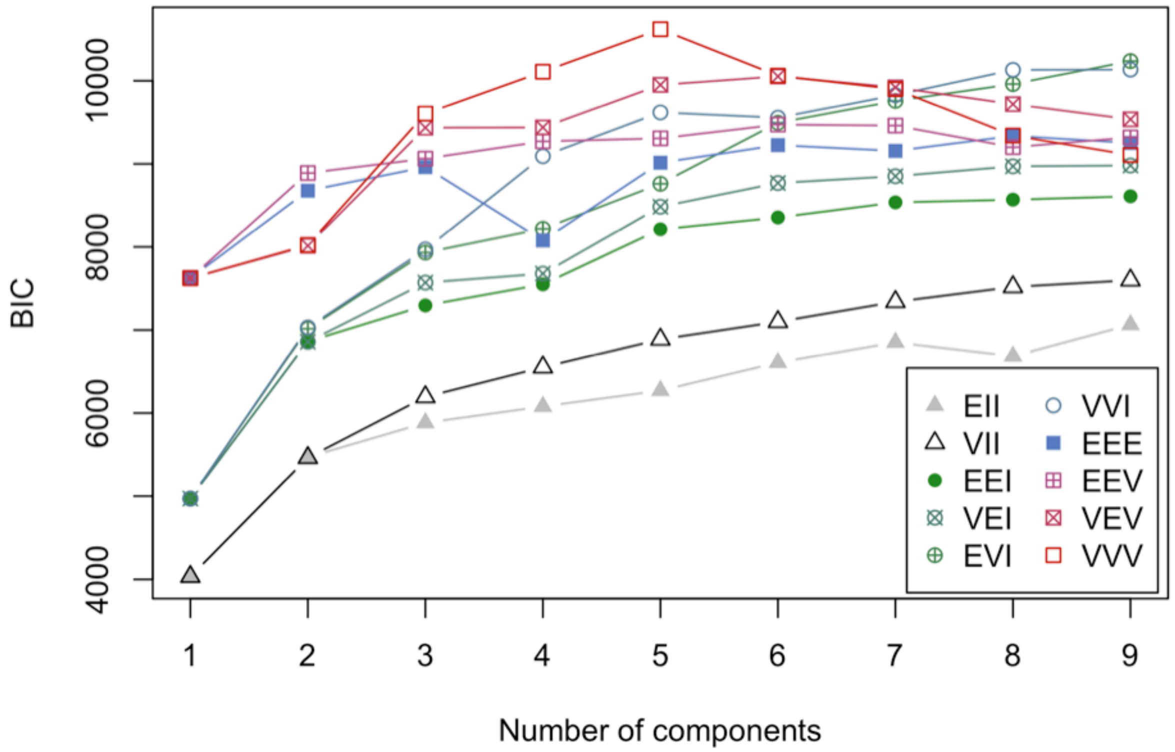

Figure S5. Distribution of study sites with time series data used to investigate temporal patterns across regimes. Map produced with ESRI ArcGIS Desktop 10.1 (http://desktop.arcgis.com/en/).

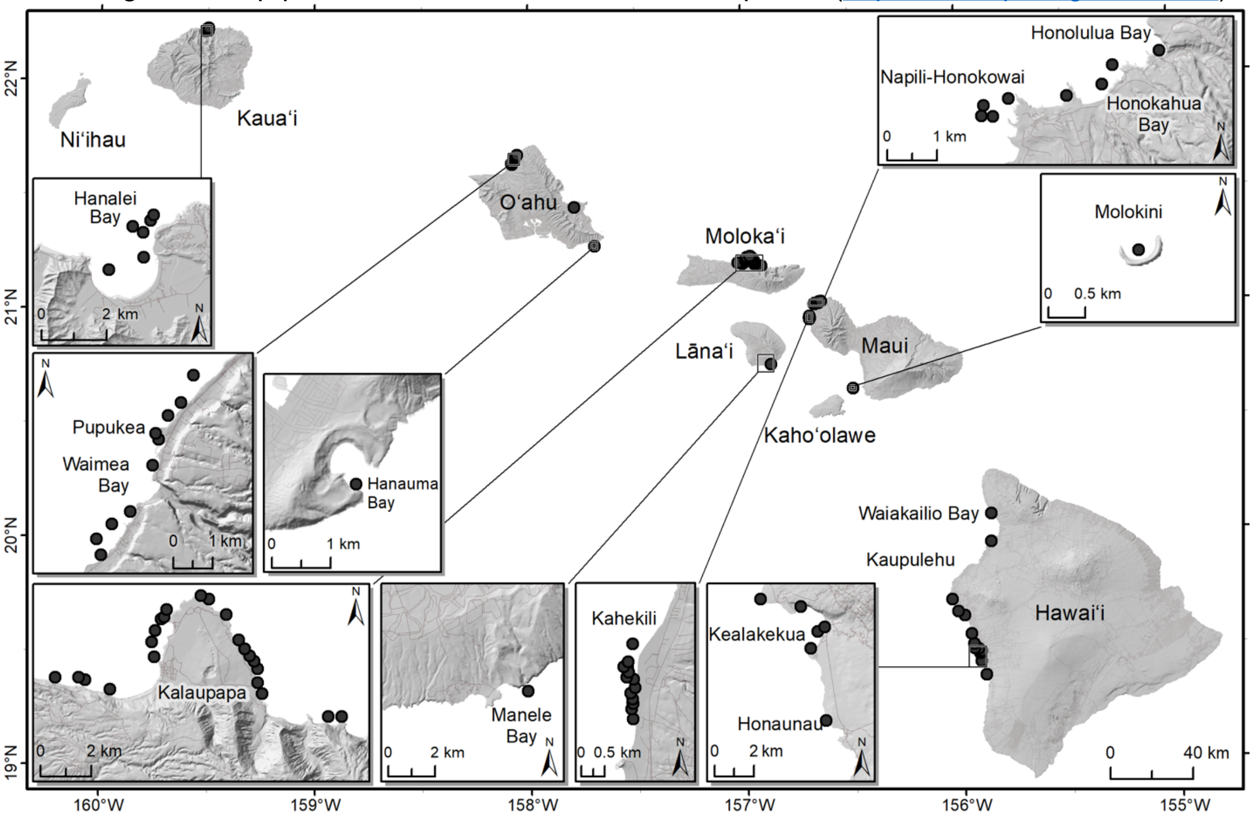

Figure S6. Comparison of binomial probabilities for each transition with 95% confidence intervals, with all 80 sites in black, 33 sites with at least 4 years of data in red, and 15 sites with at least 6 years of data in blue.

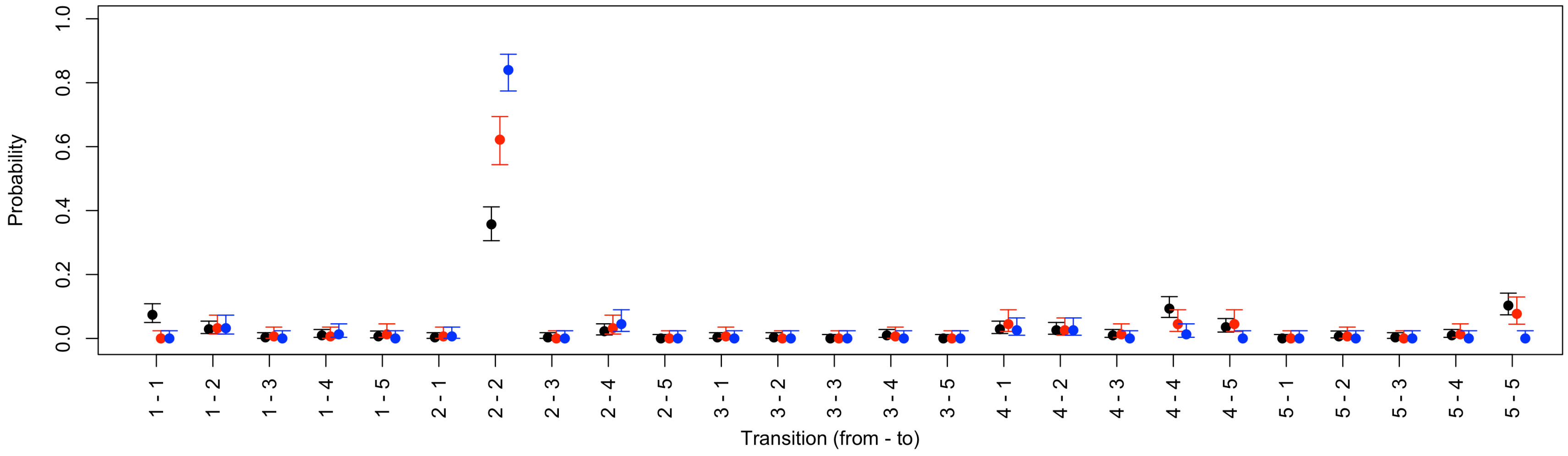

Figure S7. Examples from sites with at least 4 years of observations plotted on top of ellipses from Figure 1, colored dots and points correspond to regime (black dots used for Regime 5), and timeline on each panel. Note that some sites do not move much (e.g. Kalaupapa), while others transition through a number of defined regimes (e.g. Kahekili).

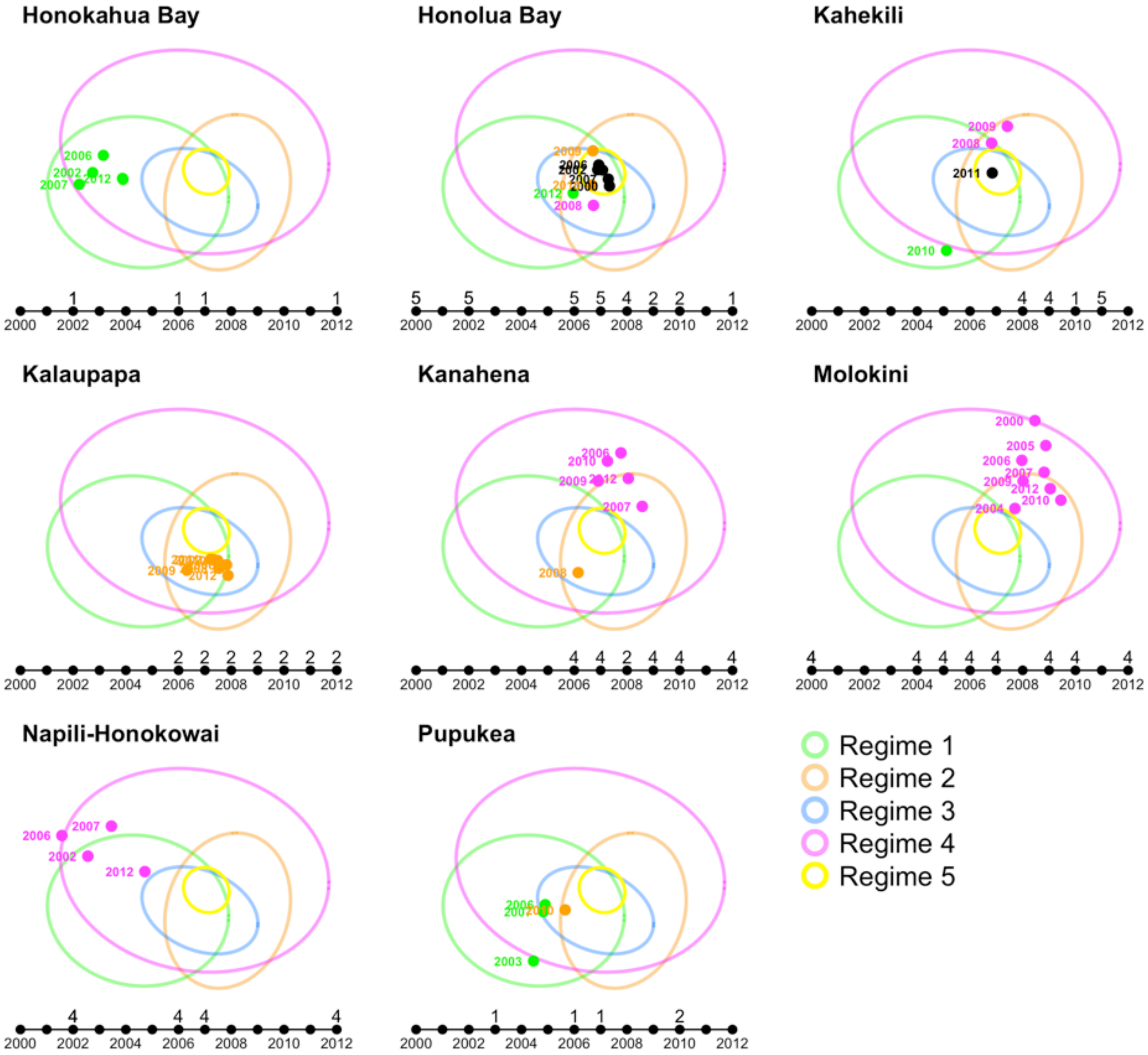

Supplementary Information: References

1. Turner, M. G., Gardner, R. H. & O'neill, R. V. *Landscape ecology in theory and practice*. **401,** (Springer, 2001).
2. Meisel, J. E. & Turner, M. G. Scale detection in real and artificial landscapes using semivariance analysis. *Landsc. Ecol.* **13,** 347–362 (1998).
3. Dale, M. R. T. & Fortin, M.-J. *Spatial analysis: a guide for ecologists*. (Cambridge University Press, 2014).
4. Diggle, P. J. & Ribeiro Jr, P. J. *Model Based Geostatistics Springer*. (Springer, 2007).
5. Ribeiro Jr, P. J. & Diggle, P. J. geoR: a package for geostatistical analysis. *R-NEWS* **1,** 15–18 (2001).
6. Plotnick, R. E., Gardner, R. H. & O'Neill, R. V. Lacunarity indices as measures of landscape texture. *Landsc. Ecol.* **8,** 201–211 (1993).
7. Fraley, C., Raftery, A. E., Murphy, T. & L, S. *mclust Version 4 for R: Normal mixture modeling for model-cased clustering, classification, and density estimation*. (Department of Statistics, University of Washington, 2012).
8. Fraley, C. & Raftery, A. E. Model-based clustering, discriminant analysis, and density estimation. *Journal of the American Statistical Association* **97,** 611–631 (2002).
9. Harrell, F. E. J. Hmisc: Harrell Miscellaneous. *https://CRAN.R-project.org/package=Hmisc* (2016).
10. Eakin, C. M. *et al.* Caribbean corals in crisis: record thermal stress, bleaching, and mortality in 2005. *PLoS One* **5,** e13969 (2010).
11. Plummer, M. rjags: Bayesian Graphical Models using MCMC. R package version 4-6. https://CRAN.R-project.org/package=rjags. (2016).
12. Gelman, A. & Rubin, D. B. Inference from iterative simulation using multiple sequences. *Stat. Sci.* 457–472 (1992).
13. Jokiel, P. L., Brown, E. K., Friedlander, A., Rodgers, S. K. & Smith, W. R. Hawai'i coral reef assessment and monitoring program: Spatial patterns and temporal dynamics in reef coral communities. *Pacific Sci.* **58,** 159–174 (2004).
14. Brown, E. K. *et al.* Development of benthic sampling methods for the Coral Reef Assessment and Monitoring Program (CRAMP) in Hawai'i. *Pacific Sci.* **58,** 145–158 (2004).
15. Friedlander, A. M., Brown, E. E., Jokiel, P. I., Smith, W. R. & Rodgers, K. S. Effects of habitat, wave exposure, and marine protected area status on coral reef fish assemblages in the Hawaiian Archipelago. *Coral Reefs* **22,** 291–305 (2003).
16. Williams, I. D. *et al.* Human, oceanographic and habitat drivers of Central and Western Pacific coral reef fish assemblages. *PLoS One* **10,** e0120516 (2015).
17. Tissot, B. N., Walsh, W. J. & Hallacher, L. E. Evaluating effectiveness of a marine protected area network in West Hawai'i to increase productivity of an aquarium fishery. *Pacific Sci.* **58,** 175–188 (2004).
18. Stamoulis, K. A., Friedlander, A. M., Meyer, C. G., Fernandez-Silva, I. & Toonen, R. J. Coral reef grazer-benthos dynamics complicated by invasive algae in a small marine reserve. *Sci. Rep.* **7,** (2017).
19. Friedlander, A. M., Brown, E. K. & Monaco, M. E. Coupling ecology and GIS to evaluate efficacy of marine protected areas in Hawaii. *Ecol. Appl.* **17,** 715–730 (2007).
20. Friedlander, A. M., Brown, E. & Monaco, M. E. Defining reef fish habitat utilization patterns in Hawaii: comparisons between marine protected areas and areas open to fishing. *Mar. Ecol. Prog. Ser.* **351,** 221–233 (2007).
